# Identifying a sufficient core group for trachoma transmission

**DOI:** 10.1371/journal.pntd.0006478

**Published:** 2018-10-08

**Authors:** Thomas M. Lietman, Michael S. Deiner, Catherine E. Oldenburg, Scott D. Nash, Jeremy D. Keenan, Travis C. Porco

**Affiliations:** 1 F.I. Proctor Foundation, University of California San Francisco, San Francisco, CA, United States of America; 2 Department of Ophthalmology, University of California San Francisco, San Francisco, CA, United States of America; 3 Department of Epidemiology & Biostatistics, University of California San Francisco, San Francisco, CA, United States of America; 4 Trachoma Control Program, The Carter Center, Atlanta, Georgia, United States of America; George Washington University School of Medicine and Health Sciences, UNITED STATES

## Abstract

**Background:**

In many infectious diseases, a core group of individuals plays a disproportionate role in transmission. If these individuals were effectively prevented from transmitting infection, for example with a perfect vaccine, then the disease would disappear in the remainder of the community. No vaccine has yet proven effective against the ocular strains of chlamydia that cause trachoma. However, repeated treatment with oral azithromycin may be able to prevent individuals from effectively transmitting trachoma.

**Methodology/Principal findings:**

Here we assess several methods for identifying a core group for trachoma, assuming varying degrees of knowledge about the transmission process. We determine the minimal core group from a completely specified model, fitted to results from a large Ethiopian trial. We compare this benchmark to a core group that could actually be identified from information available to trachoma programs. For example, determined from the rate of return of infection in a community after mass treatments, or from the equilibrium prevalence of infection.

**Conclusions/Significance:**

Sufficient groups are relatively easy for programs to identify, but will likely be larger than the theoretical minimum.

## Author summary

Public health programs can in theory target treatment to a core group of individuals responsible for a disproportionate amount of transmission. The smallest group of individuals who need to be vaccinated to eventually eliminate an infectious disease is easy to find in theory, but not in practice. While no vaccine has proven effective for trachoma, intensive periodic treatment may be able to effectively prevent individuals from transmitting infection. Here we use a variety of methods to find a core group for trachoma transmission, including methods that use information available to trachoma programs. We show that the rate that infection returns into a community after mass treatment, or the pre-treatment prevalence of an infectious disease should work, but will include more individuals than the theoretical minimum core group.

## Introduction

Core groups can play a disproportionate role in transmission of an infectious disease [1–3]. If transmission were terminated in this group, the disease would by definition eventually disappear in the entire community [1]. Important key populations have been proposed for sexually transmitted infections such as gonorrhea and HIV, as well as non-sexually transmitted diseases such as trachoma [3–10]. For vaccine-preventable diseases, the group sufficient to vaccinate to achieve herd immunity constitutes a core group. No vaccine has yet been proven effective for trachoma. However, intensive drug administration could effectively remove a core group from transmission, preventing infection from being sustainable in the remainder of the community [5].

Young age and previous infection are by far the largest risk factors for transmission of ocular chlamydia, the causative organism of trachoma [11, 12]. Children have a longer duration of infection and higher load than adults [13, 14]. In one study, repeated targeting of children reduced infection in untreated adults, suggesting a form of herd protection [15]. In another, essentially no infection could be found in adults after three years of repeated treatment of children [16]. A modeling study suggested that treating children under 10 annually could eventually eliminate infection, limiting treatment to those under 5 years might require quarterly treatments to achieve a similar result [5]. Another important risk factor is prior infection. Those with clinical or laboratory findings of trachoma at one time point are at high risk at subsequent time points, even if cleared from infection in the interim, presumably due to special vulnerability or critical placement in the transmission network [11, 17].

If all heterogeneities were identified and a mathematical transmission were completely specified, then a minimum core group could be determined analytically. However, this is rarely, if ever, feasible in practice. In this report, we hypothesize that a sufficient core group can be identified knowing very little detail about transmission. We use results from a large community-randomized trachoma trial to estimate a minimal core group for transmission. We compare several methods of estimating a sufficient core group, assuming various levels of information ranging from complete specification of the transmission dynamics to knowledge of the equilibrium prevalence alone.

## Methods

### Sufficient core group estimation methods

#### Method A, Complete specification of transmission

If we assume that the precise model is known, we can find the minimal proportion of children and adults to remove from the population to prevent a newly introduced infection from spreading. One way to model two strata would be to estimate the transmission between children and adults in a 2x2 reproduction matrix. The minimal core group would be the smallest number of children and adults necessary to remove from transmission (S1 File). We use two strata (children and adults) for demonstration. The method is applicable to any number of strata as long as the reproduction matrix is specified. While complete specification of the transmission model may be impossible in practice, here it serves as a benchmark for other methods.

#### Methods B and C, Linear Programming

If an infectious case in a homogeneous population on average causes less than one new infectious case, then infection cannot be sustained (in the absence of any positive feedback for infections). Here, we divide individuals into two homogenous groups of children and adults, and we remove enough individuals from transmission that an infected child on average infects less than one other individual (child or adult), and similarly for an adult. This would ensure that those removed form a sufficient core group, and that the overall *R* for the community is less than one [1]. The goal is always feasible, as long as enough individuals are removed from transmission. Knowledge of the reproduction matrix allows this to be formulated as a linear programming problem, maximizing a linear function given a set of linear inequality constraints. A linear program has a dual solution, which is also core group (C) (see S1 File) [18]. If both the primal and the dual have solutions that include both children and adults, they necessarily sum to the same total number of individuals (Duality Theorem) [18]. These methods require that we know that no individual is expected to successfully transmit infection to more than one other person, which would be difficult to determine in practice. In general, neither of the solutions is optimal.

#### Method D. Rate of return of infection into a community following mass treatment

In practice, we typically know little about the heterogeneities in transmission, and certainly not enough to accurately estimate components of a next generation *R* matrix. However, studies have estimated the rate that infection returns into a community after mass antibiotic distribution [5, 19]. If combined with knowledge of the average duration of infection, we can estimate a growth rate per generation of infection, which is closely related to the maximum eigenvalue (*λ*) of the *R* matrix, and suggests a method for ensuring a sufficient core group. If we remove from transmission at least the proportion 1-1/*λ* of each stratum, then infection can no longer be sustained in the community (see S1 File) [5, 13, 19, 20]. Note that for a given rate of return, a longer duration of infection would increase the estimate of the size of the core group.

#### Method E. Equilibrium

In trachoma programs a pre-treatment survey is often performed before programmatic activity. If conditions are relatively stable, then this prevalence represents an estimate of the equilibrium. By definition, at equilibrium each infectious case causes on average a single new infectious case. If we were to remove from transmission at least the equilibrium proportion for each stratum of the population, then infection would no longer be sustainable. Here we implement with two strata, although this approach applies in general to any number of strata.

In some areas of Ethiopia, up to 10 years of annual mass drug administrations have failed to completely eliminate infection [21]. The prevalence of clinical signs has been reduced but over the past several years remained relatively stable, suggesting a new, reduced equilibrium. This new restricted equilibrium could also represent a sufficient core group, although only if annual mass distributions continued.

### Three scenarios modeled

To explore the 5 different methods for determining a sufficient core group under different conditions, we modeled 3 possible scenarios:

#### Scenario I

To mimic a region hyperendemic for trachoma before any programmatic activity, we used a simple susceptible-infectious-susceptible (SIS) model that had previously been fitted to results from a cluster-randomized trial in Amhara, Ethiopia (TANA, NEI U10 EY016214) [15, 16, 22]. Infection was monitored in 0–10 year olds (“children”) and in those older than 10 years (“adults”). Estimates of the transmission between children and adults had previously been made, defining estimated reproduction matrix for children and adults. This model can be used to compare 5 methods (A-E) of determining a sufficient core group (see S1 File) [15, 16, 22].

#### Scenario II

In some areas hyperendemic for trachoma, a decade of annual mass treatment has failed to eliminate infection, resulting in a new reduced equilibrium [21]. We have constructed a reproduction matrix which is reduced from Scenario I due to the continued annual mass treatment, assuming annual antibiotic coverage for 5 years.

#### Scenario III

To demonstrate how the possible core groups would change if both children and adults were substantial contributors to transmission, we constructed a hypothetical community in which members of both the children and adult segments of the population could themselves sustain infection. Note that members from each stratum would need to be included in any core group.

## Results

The total proportion of children and adults that form a core group was determined using each of the 5 methods, for each of the three scenarios (Table 1). For explanatory purposes, the total proportion in the population was estimated assuming that children and adults were each one half of the population, although it is not difficult to adjust this ratio. Note that approximately half the population in these areas may be 18 years and under, one third 10 years and under, and one-sixth under the age of 5 years.

**Table 1:** Results for 3 scenarios modeled.

Scenario	R Matrix		Total proportion of population	
I Hyperendemic for trachoma, Pretreatment	1.39	0.67	A	0.32
			B	0.47
	1.48	0.28	C	0.64
			D	0.49
			E	0.48
II Hyperendemic for trachoma, Postreatment	0.95	0.38	A	0.15
			B	0.19
	0.83	0.33	C	n/a
			D	0.22
			E	0.21
III Children and Adults	1.45	0.25	A	0.45
			B	0.46
	0.48	1.52	C	0.46
			D	0.45
			E	0.45

For each of three scenarios I-III modeled, the R matrix is given in the center column. The 4 cells of each R matrix reveal the average number of secondary infectious cases caused by a single infectious case in a totally susceptible population. The upper left is the number of child cases caused by a single child case, the upper right child cases caused by an adult case, lower left adult cases caused by a child case, and lower right adult cases caused by an adult case. Within each scenario, the total proportion of the population for the 5 models A-E is also presented (A. Complete specification of transmission, B. Primal Linear Programming, C. Dual Linear Programming, D. Largest Perron eigenvalue, E. Equilibrium).

### Scenario I

**Fig 1:**
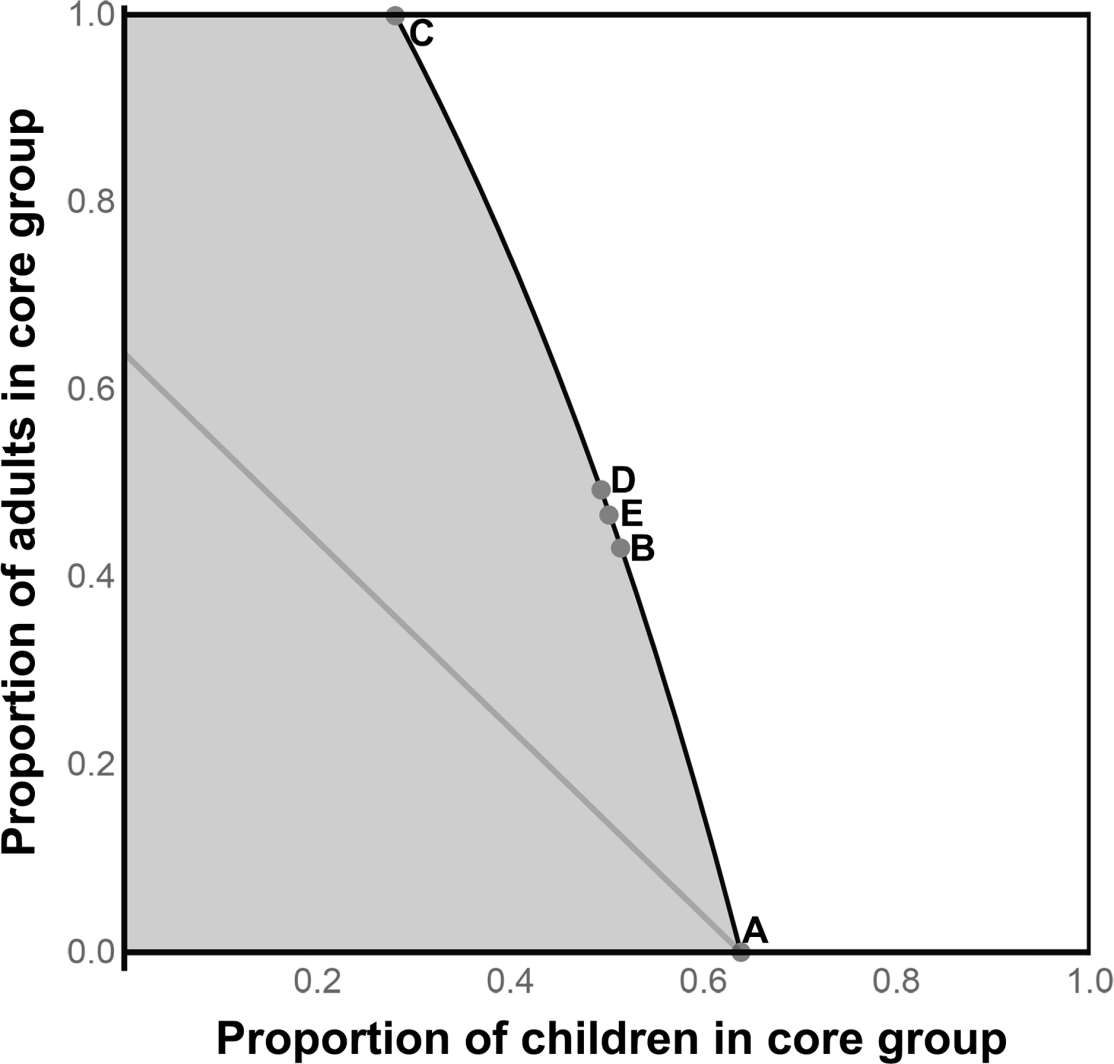
An area hyperendemic for trachoma (Scenario I). The x-axis is the proportion of children that are in a potential core group, and the y-axis is the proportion of adults. The black curve represents those proportions where the resulting reproduction number (R) of transmission in the entire population equals unity. If the proportion of children and adults represented by any combination above and to the right of this curve (the white, non-shaded area) were removed from transmission, infection could not be sustained in the rest of the population. Thus, all combinations in the white area represent sufficient core groups. Point A represents the minimal core group whose removal would result in eventual elimination of infection. Point B and C represent sufficient core groups determined by linear programming, point D from the maximum eigenvalue of the transmission matrix (estimated directly from the return of infection into a community and the average duration of infection), and point E from the equilibrium, pre-treatment prevalence of infection.

Given a transmission model estimated from the TANA study, the theoretical minimum core group includes 64% of the children and none of the adults for a total of 32% of the total population (Fig 1 and Table 1). Sufficient core groups using linear programming, the maximum eigenvalue, or the equilibrium prevalence included more than twice the total number of individuals.

### Scenario II

**Fig 2:**
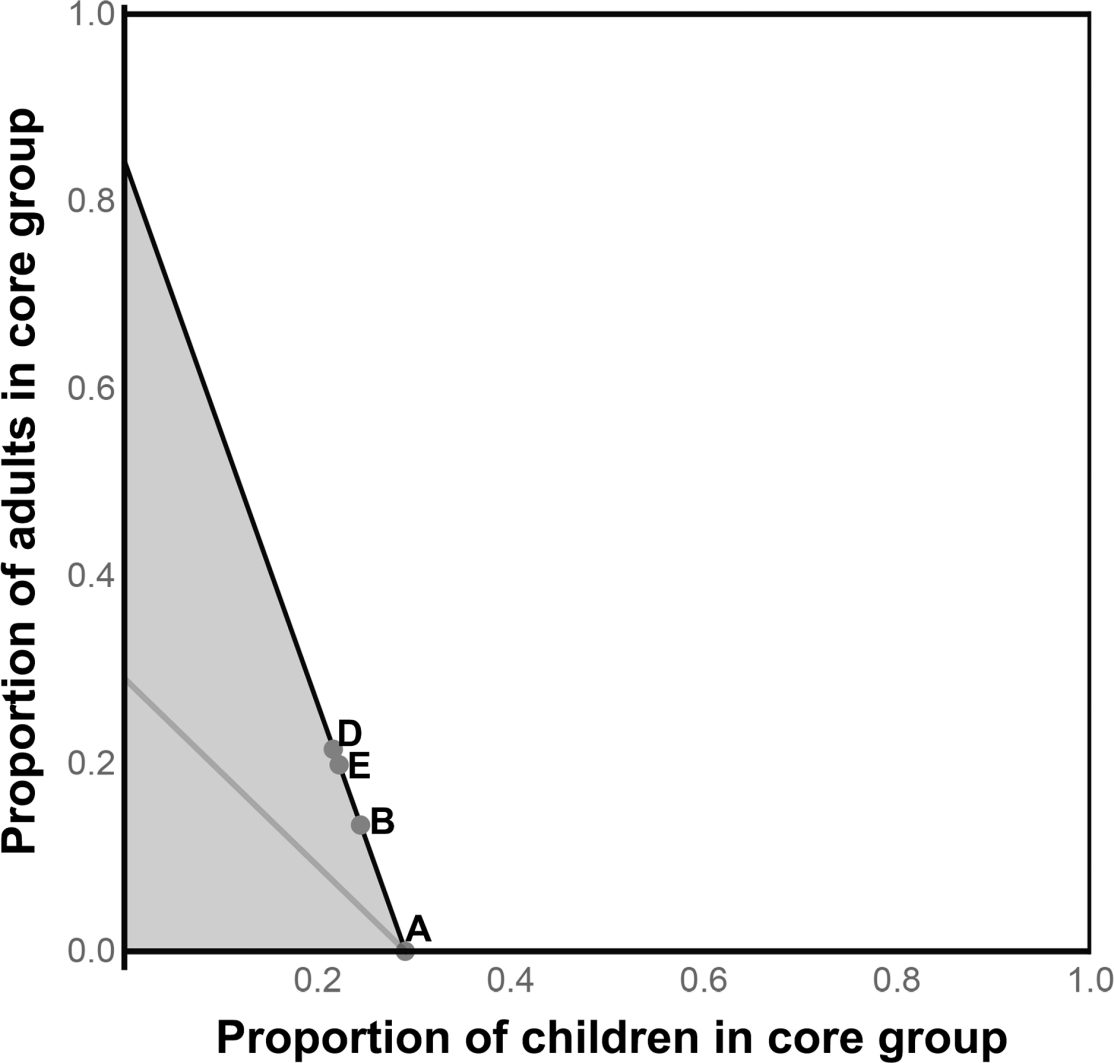
After a decade of annual mass antibiotic distributions (Scenario II). Infection has still not been eliminated in some areas of Ethiopia. The prevalence of infection has reached a new, residual equilibrium in the presence of the continued treatments. The residual core group is smaller than the core group in Scenario I. If this group were targeted and mass treatments were continued, we would expect infection to eventually disappear. The minimal core group (A) includes only children, but the strategies based on the rate of return of infection (D) and the residual equilibrium (E) may offer practical, reasonably efficient strategies. The dual linear programming solution is not feasible here, and is not represented.

In the presence of years of mass annual treatment, a new equilibrium may be reached. Here, 21% of children and adults remain affected. A residual core group remains, in the presence of the annual treatments (Fig 2, Table 1). The sufficient core groups obtained from the rate of return of infection and from the residual equilibrium are still larger as the theoretical minimum of 15%, including 21–22% of the population.

### Scenario III

**Fig 3:**
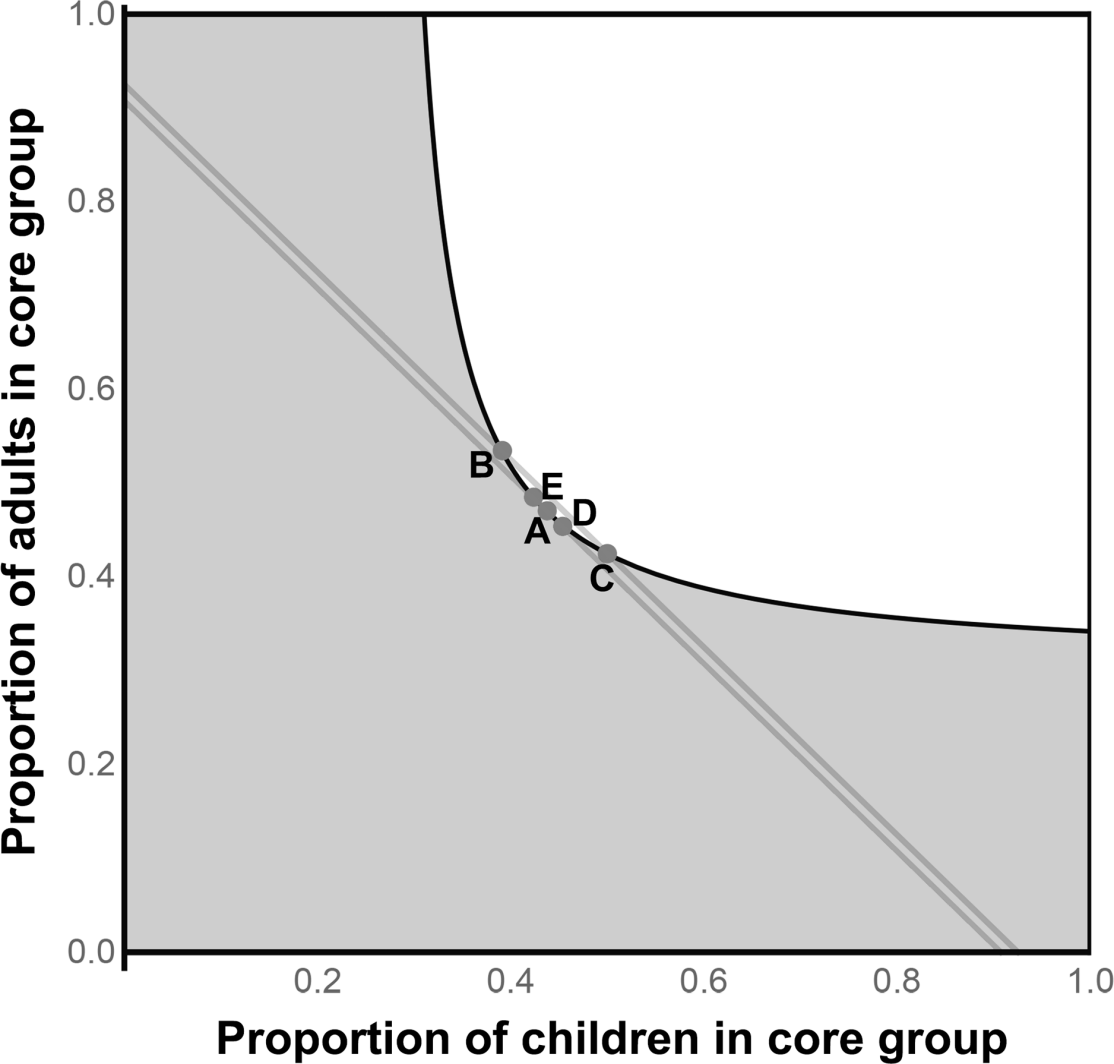
A hyper-endemic community where transmission can be sustained in either the children alone or the adults alone (Scenario III). Here, all solutions are similar to the theoretical minimum core group (A). The primal and dual linear programming solutions contain different combinations of children and adults, but contain the same overall total [18].

In the most severely affected areas, transmission can be sustained in either the children alone or the adults alone. Therefore, all sufficient core groups would necessarily include both children and adults. Here, the more practical methods approach the theoretical minimum (Fig 3, Table 1). In this scenario, both the primal (B) and dual (C) linear programs have identified core groups that contain both children and adults. Although the combinations are different, they contain the same number of individuals as demonstrated by the second (higher) grey iso-population line, as guaranteed by the Duality Theorem [18]. Note that transmission in adults in this scenario is likely unrealistically high, although we have included it as an illustrative example.

## Discussion

A group of individuals who play a disproportionate role in the transmission of an infectious disease has been described by a number of terms, including key population, hyper-spreaders, and core group [10, 23]. A stricter definition of a core group would be those individuals whose removal from transmission would be sufficient to prevent infectious trachoma from sustaining itself in the entire community [1, 24]. Here, we have assumed that the process can be described by a mathematical transmission model in which individuals can be divided into a number of compartments, each one of which is homogeneous. From such a model, a sufficient core group can be estimated in a number of ways. As a relatively simple case, we divide a trachoma-endemic population into children and adults, and fit a simple model to data from a community-randomized trial in Ethiopia [15]. The fitted model allows a sufficient core group to be estimated using a number of methods. The smallest group whose removal from transmission results in the remainder of the population producing on average less than one case per infectious case. Under certain circumstances, the dual of this linear programming problem also may be a sufficient core group. If the dominant eigenvalue of a next generation matrix of the transmission model and the average duration of infectious cases in each of the groups are defined, then a sufficient core group can easily be determined. If the entire model is specified, then the absolute minimum sized core group can be found.

Even when the precise details of the transmission model are not understood, a sufficient core group can still be estimated from epidemiological data. If we limit analysis to diseases that do not confer lifelong immunity and where current infection is a risk factor for future reinfection, then removal of those currently infected from future transmission would control the disease, leading to eventual elimination. For example, if infection is reduced by repeated mass antibiotic distributions given to the entire community, then it should return at a rate dependent on the maximum eigenvalue of the next generation matrix. If the average duration of infection is known, then the dominant eigenvalue of this matrix can be estimated. If all but the proportion equal to the reciprical of this eigenvalue were removed from each compartment, then infection would eventually disappear.

A more practical method for determining a sufficient core group requires knowledge of who was infected prior to treatment. If we assume that current infection is a risk factor for future reinfection. This allows determination of a core group in two settings: at the beginning of a program when transmission may have reached an equilibrium, and after years of annual MDA with a new equilibrium. In the former, the goal would be to find the group of people whose effective removal from transmission would lead to eventual elimination in everyone. Community-randomized trials have found that if a core group of children are effectively removed from transmission by quarterly antibiotic treatments, infection decreases in the remainder of the community [15]. If mass antibiotics are repeated, elimination can be achieved even though not everyone was actually treated. In the setting where years of annual mass treatment have not eliminated infection, then the goal might be to find a residual core group whose removal from transmission would result in elimination if future annual mass treatment were also continued. In several areas, more than 5 years of mass azithromycin distributions have left a small amount of persistent infection [25, 26]. This group could represent a residual core group.

The equilibrium approach to finding a sufficient core group is dependent on a number of assumptions. For the equilibrium to form a core group, currently infected cases must be at least as likely to participate in future transmission as uninfected cases. This would not be true for an infection where acquired immunity outweighs other risk factors. Note that removing those infected at equilibrium from transmission would be expected to work regardless of whether we understand the heterogeneity in the community. Underestimates of prevalence would lead to models underestimating the size of the core group. We modeled 3 scenarios of different endemicity, reflecting different transmission. Future study could explore scenarios with an even broader spectrum of heterogeneity. While method 1 assumed an SIS model structure, that is not necessary for the other methods. However, we have assumed that current infection is a risk factor for future infection, or at least does not offer protection. If this assumption were violated, more analysis would be necessary.

Sufficient core groups can be estimated from information available to trachoma control programs. The use of WHO simplified grades rather than infection data would result in a larger, more conservative estimate of the core group. In moderately affected areas, information taken from baseline surveys could be used to reduce the proportion of the population treated. In the few severely affected areas where residual core group indicates that annual MDA is inadequate, enhanced targeting of a core group might be necessary for elimination. Enhanced intervention targeted to core groups would require additional resources, but may be the final key for successful trachoma control. Resources can be directed towards core groups at the beginning of a program, or towards a residual core groups resistant to elimination in the presence of standard annual mass azithromycin distribution [21]. Precise transmission models are not absolutely necessary to estimate sufficient core groups. These models are hypothesis generating. While strategies based on core groups have been tested in community-randomized trials, the efficacy of a strategy based on residual core groups will need to be tested in current and future trials (U10 EY023939 and UG1 EY028088) [15, 16, 27].

## Supporting information

S1 File **Methods A-E.** Additional details are provided for Methods A-E.
(PDF)

## References

[1] Jacquez J, Simon C, Koopman J. Core groups and R_0_s for subgroups in heterogeneous SIS and SI models In: Mollison D, editor. Epidemic models: Their Structure and Relation to Data. Cambridge: Cambridge University Press; 1995 p. 279–301.

[2] Anderson R, May R. Infectious Diseases of Humans: Dynamics and Control Oxford: Oxford University Press; 1991.

[3] Hethcote HW, Yorke JA. Gonorrhea transmission dynamics and control Berlin: Springer-Verlag; 1984.

[4] Hethcote H. Modeling heterogeneous mixing in infectious disease dynamics In: Isham V, Medley G, editors. Modeling Heterogeneous Mixing in Infectious Disease Dynamics Models for Infectious Human Diseases: Their Structure and Relation to Data. Cambridge: Cambridge University Press; 1996 p. 215–38.

[5] Lietman T, Porco T, Dawson C, Blower S. Global elimination of trachoma: how frequently should we administer mass chemotherapy? Nature Medicine. 1999;5(5):572–6. 10.1038/8451 10229236

[6] Gesink DC, Sullivan AB, Miller WC, Bernstein KT. Sexually transmitted disease core theory: roles of person, place, and time. Am J Epidemiol. 2011;174(1):81–9. 10.1093/aje/kwr035 21540320 PMC3159428

[7] Watts C, Zimmerman C, Foss AM, Hossain M, Cox A, Vickerman P. Remodelling core group theory: the role of sustaining populations in HIV transmission. Sex Transm Infect. 2010;86 Suppl 3:iii85–92. 21098061 10.1136/sti.2010.044602

[8] Lewis DA. The role of core groups in the emergence and dissemination of antimicrobial-resistant N gonorrhoeae. Sex Transm Infect. 2013;89 Suppl 4:iv47–51. 24243880 10.1136/sextrans-2013-051020

[9] Boily MC, Shubber Z. Modelling in concentrated epidemics: informing epidemic trajectories and assessing prevention approaches. Curr Opin HIV AIDS. 2014;9(2):134–49. 10.1097/COH.0000000000000036 24468893

[10] UNAIDS. UNAIDS Terminology Guidelines. http://www.unaids.org/sites/default/files/media_asset/2015_terminology_guidelines_en.pdf: UNAIDS; 2015. Contract No.: February 14.

[11] Schachter J, West SK, Mabey D, Dawson CR, Bobo L, Bailey R, et al Azithromycin in control of trachoma. Lancet. 1999;354(9179):630–5. 10.1016/S0140-6736(98)12387-5 10466664

[12] Bird M, Dawson CR, Schachter JS, Miao Y, Shama A, Osman A, et al Does the diagnosis of trachoma adequately identify ocular chlamydial infection in trachoma-endemic areas? J Infect Dis. 2003;187(10):1669–73. 10.1086/374743 12721948

[13] Bailey R, Duong T, Carpenter R, Whittle H, Mabey M. The duration of human ocular Chlamydia trachomatis infection is age dependent. Epidemiology and Infection. 1999;123(3):479–86. 10694161 10.1017/s0950268899003076 PMC2810784

[14] Solomon AW, Holland MJ, Alexander ND, Massae PA, Aguirre A, Natividad-Sancho A, et al Mass treatment with single-dose azithromycin for trachoma. N Engl J Med. 2004;351(19):1962–71. 10.1056/NEJMoa040979 15525721 PMC6850904

[15] House JI, Ayele B, Porco TC, Zhou Z, Hong KC, Gebre T, et al Assessment of herd protection against trachoma due to repeated mass antibiotic distributions: a cluster-randomised trial. Lancet. 2009;373(9669):1111–8. 10.1016/S0140-6736(09)60323-8 19329003

[16] Amza A, Kadri B, Nassirou B, Cotter SY, Stoller NE, Zhou Z, et al A Cluster-Randomized Trial to Assess the Efficacy of Targeting Trachoma Treatment to Children. Clinical infectious diseases: an official publication of the Infectious Diseases Society of America. 2017;64(6):743–50. 27956455 10.1093/cid/ciw810

[17] West SK, Muñoz B, Lynch M, Kayongoya A, Mmbaga BB, Taylor HR. Risk factors for constant, severe trachoma among preschool children in Kongwa, Tanzania. American Journal of Epidemiology. 1996;143(1):73–8. 8533749 10.1093/oxfordjournals.aje.a008659

[18] Franklin J. Methods of Mathematical Economics First ed. New York: Springer-Verlag New York Inc; 1980 1980.

[19] Melese M, Chidambaram JD, Alemayehu W, Lee DC, Yi EH, Cevallos V, et al Feasibility of eliminating ocular Chlamydia trachomatis with repeat mass antibiotic treatments. JAMA. 2004;292(6):721–5. 10.1001/jama.292.6.721 15304470

[20] Grassly NC, Ward ME, Ferris S, Mabey DC, Bailey RL. The natural history of trachoma infection and disease in a gambian cohort with frequent follow-up. PLoS Negl Trop Dis. 2008;2(12):e341 10.1371/journal.pntd.0000341 19048024 PMC2584235

[21] WHO. Meeting of the International Task Force for Disease Eradication, November 2016. Weekly epidemiological record. 2017;92(9/10):106–16. 28262011

[22] Ying R, Williams PD, Dorratoltaj N, Sempa J, Lourens L, Liu F, et al. Prospects for trachoma elimination through mass treatment targeted at children. Proceedings of the Agent-Directed Simulation Symposium: Society for Computer Simulation; 2016.

[23] Jacquez JA, Simon CP. The stochastic SI model with recruitment and deaths. I. Comparison with the closed SIS model. Math Biosci. 1993;117(1–2):77–125. 8400585 10.1016/0025-5564(93)90018-6

[24] Diekmann O, Heesterbeek JA, Metz JA. On the definition and the computation of the basic reproduction ratio R0 in models for infectious diseases in heterogeneous populations. J Math Biol. 1990;28(4):365–82. 2117040 10.1007/BF00178324

[25] Melese M, Alemayehu W, Lakew T, Yi E, House J, Chidambaram JD, et al Comparison of annual and biannual mass antibiotic administration for elimination of infectious trachoma. Jama. 2008;299(7):778–84. 10.1001/jama.299.7.778 18285589

[26] Gebre T, Ayele B, Zerihun M, Genet A, Stoller NE, Zhou Z, et al Comparison of annual versus twice-yearly mass azithromycin treatment for hyperendemic trachoma in Ethiopia: a cluster-randomised trial. Lancet. 2012;379(9811):143–51. 10.1016/S0140-6736(11)61515-8 22192488

[27] Holm SO, Jha HC, Bhatta RC, Chaudhary JS, Thapa BB, Davis D, et al Comparison of two azithromycin distribution strategies for controlling trachoma in Nepal. Bulletin of the World Health Organization. 2001;79(3):194–200. 11285662 PMC2566365

