# Supplementary material for: Identifying a sufficient core group for trachoma transmission
Source: PLoS Negl Trop Dis. 2018 Oct 8;12(10):e0006478. doi: 10.1371/journal.pntd.0006478 (PMC6175502; doi:10.1371/journal.pntd.0006478)
Supplement: S1 File — Methods A-E. Additional details are provided for Methods A-E. (PDF) [file pntd.0006478.s001.pdf]

Supporting Information S1

Method A. Complete specification of transmission. We assumed that the population was divided into 2 homogeneous subgroups (strata), in this case children and adults. We represent the dynamics by a discrete time (generations of infection) model. Specifically, let t denote the generation, let $\mathbf{x}(t)$ denote the vector of the fraction of susceptibles in each stratum, let $\mathbf{y}(t)$ denote the vector of the fraction of infectious cases in each stratum, and let $\mathbf{R_0}$ denote the next generation reproduction matrix. Denoting the fraction in class i removed from transmission by z_i (elements of the vector $\mathbf{z}$), we have $\mathbf{x}(t)=1-\mathbf{y}(t)-\mathbf{z}$, and we can write

$$\mathbf{y}(t+1) = \mathbf{diag}(\mathbf{x}(t))\cdot\mathbf{R_0}\cdot\mathbf{y}(t)$$

The infection will not be sustainable in the community if the maximum eigenvalue of the matrix $diag(\mathbf{x}(t))\cdot R_0$ is less than one (where $\mathbf{diag}(\mathbf{x})$ is a diagonal matrix whose i,i-th element is x_i). Thus, we solve for the minimal infectious removed from the community $\sum_i z_i$ such that the maximum eigenvalue of $diag(\mathbf{x}(t))\cdot R_0$ is equal to one.

Methods B and C. Linear Programming. Elimination can be assured if each individual infectious case causes on average fewer than one new infectious case. This is a sufficient but not necessary condition, provided no positive feedback for infectives exists. Each individual causes on average fewer than one case when we remove enough individuals from each stratum so that every row $R_{i.}$ of $R=diag(1-z)\cdot R_0$ satisfies $\sum_j R_{ij}<1$. Minimizing z allows estimation of the minimal group of individuals whose removal ensures that all rows are less than one. Thus the principal eigenvalue would necessarily be less than one. Note that this identified core group may not be the *minimum* core group, as it is possible to have a principal eigenvalue less than one even if one of the rows sums to greater than one. Similarly, a sufficient core group can be obtained by ensureing that the column sums of the adjust matrix are less than one: $R= R_0\cdot diag(1-z)$ and $\sum_i R_{ij}<1$. These two solutions can be obtained by straightforward linear programming, with the constraint that $0\leq z\leq 1$. If the first solution is set up as the primal and the second the dual, the duality theorem guarantees that if both have feasible solutions which include children and adults in the core group, that the total number of individuals would be the same, although the combination of children and adults may differ. If no such solutions exist, then there can be a duality gap between the primary and dual.

Method D. Largest (Perron) eigenvalue. In practice, we typically know little about the heterogeneities in transmission, and certainly not enough to accurately estimate components of an R matrix. But we have been able to estimate the rate that infection returns into a community after multiple mass distributions. The maximum eigenvalue can be estimated from the rate of return of infection and the average duration of infection.[5,8,14,15] Any combination of individuals taken out of the children and adult strata that results in the maximum eigenvalue of the matrix $diag(1-z_t)\cdot R_0$ being less than one would be a core group. If we remove $1-1/\lambda$, resulting in both children and adult susceptibles of $x = (1/\lambda\ , 1/\lambda)$, the maximum eigenvalue will necessarily be one.

These allow estimation of the R_0 matrix's dominant eigenvalue λ. If we remove a proportion of individuals from each stratum reciprocal to this eigenvalue λ, the proportion of children and adult strata that results ensure that infection cannot return into a community post treatment (ie, the maximum eigenvalue of the new matrix $\boldsymbol{diag(1\text{-}z_t)\cdot R_0}$ equals one).

Method E. Equilibrium. More readily available may be the equilibrium prevalence. For example, in trachoma programs a pre-treatment survey is often performed before programmatic activity, and this represents an estimate of the equilibrium. One feasible core group would be more than those infected at equilibrium (the fraction susceptible at equilibrium is one minus the fraction infected), as this would ensure that the principal eigenvalue of the matrix $\boldsymbol{diag(1\text{-}z_t)\cdot R_0}$ would be one. After repeated annual distributions of antibiotic, some areas in Ethiopia have reached a new equilibrium. This new equilibrium could represent a sufficient core group, in the setting of annual distributions. If transmission could be curtailed in this residual core group and mass distributions continued, then we would expect infection to eventually disappear in the entire community.
